# Host immunity associated with spontaneous suppression of viremia in therapy-naïve young rhesus macaques following neonatal SHIV infection

**DOI:** 10.1128/jvi.01094-23

**Published:** 2023-10-24

**Authors:** Tyler D. Evangelous, Madison Berry, Sravani Venkatayogi, Cas LeMaster, Eric S. Geanes, Nicole De Naeyer, Todd DeMarco, Xiaoying Shen, Hui Li, Bhavna Hora, Nicholas Solomonis, Johnathan Misamore, Mark G. Lewis, Thomas N. Denny, David Montefiori, George M. Shaw, Kevin Wiehe, Todd Bradley, Wilton B. Williams

**Affiliations:** 1 Duke Human Vaccine Institute, Duke University School of Medicine, Durham, North Carolina, USA; 2 Children’s Mercy Kansas City, Kansas City, Missouri, USA; 3 Department of Surgery, Division of Surgical Sciences, Duke University School of Medicine, Durham, North Carolina, USA; 4 Perelman School of Medicine, University of Pennsylvania, Philadelphia, Pennsylvania, USA; 5 BIOQUAL Inc., Rockville, Maryland, USA; 6 Department of Pediatrics, UMKC School of Medicine, Kansas City, Missouri, USA; 7 Departments of Pediatrics and Pathology, University of Kansas Medical Center, Kansas City, Kansas, USA; 8 Department of Integrative Immunobiology, Duke University School of Medicine, Durham, North Carolina, USA; Ulm University Medical Center, Ulm, Baden-Württemberg, Germany

**Keywords:** cytokine profiling, single-cell transcriptomics, macaque BCR sequencing, macaque TCR sequencing, neonatal SHIV infection, viremia control, viral persistence

## Abstract

We recently found that a new pathogenic chimeric simian-human immunodeficiency virus (SHIV) elicited heterologous human immunodeficiency virus type-1 (HIV-1) neutralizing antibodies (nAbs) in therapy-naïve young rhesus macaques (RMs) following neonatal SHIV infection. Moreover, a subset of the SHIV-infected young RMs spontaneously controlled viremia. Here we evaluated humoral and cellular immunity and plasma biomarkers associated with spontaneous viremia suppression in a new model of young SHIV-infected RMs that generated heterologous HIV-1 nAbs independent of viremia control to gain insights into pediatric immunity that may be harnessed by appropriate therapies in HIV-1-infected infants and children. We determined the levels of 31 plasma analytes (cytokines, chemokines, and growth factors) in SHIV-infected RMs over the course of infection and found that six analytes with chemoattractant or pro-inflammatory activities had significantly lower levels in plasma of RMs that controlled viremia compared to non-controllers. Single-cell transcriptomics of blood-derived immune cells demonstrated that RMs with viremia control had upregulated genes associated with immune activation and cytotoxic functions, whereas non-controllers had upregulated genes associated with immune cell exhaustion and dysfunction. In addition to CD8 T and natural killer cells, monocytes with upregulation of inhibitory genes previously reported only in cytotoxic cells constituted the immunologic environment associated with viremia suppression. These data implicated a complex immunologic milieu of viremia suppression that is not fully defined in pediatric subjects. Understanding immune cell subsets that may be harnessed to control viremia will provide insights into future designs of HIV-1 therapeutic strategies.

**IMPORTANCE:**

Despite the advent of highly active anti-retroviral therapy, people are still dying from HIV-related causes, many of whom are children, and a protective vaccine or cure is needed to end the HIV pandemic. Understanding the nature and activation states of immune cell subsets during infection will provide insights into the immunologic milieu associated with viremia suppression that can be harnessed via therapeutic strategies to achieve a functional cure, but these are understudied in pediatric subjects. We evaluated humoral and adaptive host immunity associated with suppression of viremia in rhesus macaques infected soon after birth with a pathogenic SHIV. The results from our study provide insights into the immune cell subsets and functions associated with viremia control in young macaques that may translate to pediatric subjects for the design of future anti-viral strategies in HIV-1-infected infants and children and contribute to an understudied area of HIV-1 pathogenesis in pediatric subjects.

## INTRODUCTION

Human immunodeficiency virus type-1 (HIV-1) infects lymphoid and myeloid immune cells (1, 2) and establishes viral reservoirs soon after infection; thus, eradication of viral reservoirs is paramount to achieving a cure (3 – 5). CD4 T lymphocytes are the primary targets for infection and viral reservoirs (6, 7), and cytolytic CD8 T cells and natural killer (NK) cells play key roles in eliminating HIV-1-infected cells (8 – 10). However, chronic immune activation is a hallmark of HIV-1 infection that leads to immune cell dysfunction and exhaustion or senescence, and has been postulated to be a cause of ineffective host immunity in controlling viremia and disease progression to acquired immune deficiency syndrome (AIDS) (11 – 14). Understanding the nature and activation states of immune cells in the immunologic milieu associated with spontaneous viremia suppression in the absence of combined anti-retroviral therapy (cART) will inform appropriate therapeutic strategies to harness them (15).

We recently described heterologous HIV-1 neutralizing antibodies (nAbs) that have the potential to develop breadth as broadly reactive nAbs (bnAbs) in rhesus macaques (RMs) following neonatal infection with a new pathogenic subtype C simian-human immunodeficiency virus (SHIV) (16). Additionally, we found that a subset of young SHIV-infected RMs suppressed viremia. Thus, we now have a new SHIV-infected pediatric animal model that can be exploited to identify host immunity associated with viremia suppression, which is understudied in HIV-1-infected infants and children. Vieira and colleagues demonstrated that robust HIV-specific CD4+ T cells, in addition to CD8+ T cells, modulate elite control of HIV-1 in adolescents (17), and studies in adult humans have reported circulating or tissue-specific cytolytic CD8 T cells as the primary driver for spontaneous viremia control (18 – 20). However, other studies have implicated a role for innate immune cells in spontaneous viremia control following HIV-1 infection (21), suggestive of a complex immunologic milieu associated with spontaneous HIV-1 suppression.

In this study, we evaluated host immunity associated with partial or full control of viremia in cART-naïve young RMs infected with a pathogenic SHIV that was recently described (16). Longitudinal plasma viral load and HIV-1 nAb responses following neonatal SHIV infection in young RMs demonstrated the durability of neonatal immunity to spontaneously control viremia and/or generate HIV-1 nAbs after 3 years of therapy-naïve SHIV infection, which has never been reported. Plasma analyte profiling, including cytokines, chemokines, and growth factors, revealed that viremia suppression was associated with a less inflammatory milieu. Using our recently established single-cell immune profiling assay (22), we evaluated the transcriptome of RM immune cells, as well as the B- and T-cell receptor (TCR) sequences, to understand the characteristics, function, and ontogeny of immune cell subsets during viremia suppression. In agreement with previous studies of elite HIV-1 controllers, we identified CD8-expressing cells, including T and NK cells, and varied CD4 T-cell subsets that were associated with viremia suppression in young SHIV-infected RMs. We also identified intermediate monocytes with upregulation of inhibitory genes previously described in cytotoxic cells as new candidate innate immune cells that constitute the immunologic milieu of viremia control in young SHIV-infected RMs. Thus, we used a recently established pediatric macaque model (16) to gain further insights into immunologic factors associated with viremia suppression that may be exploited for appropriate therapeutic strategies in HIV-1 infected infants and children.

## RESULTS

### Pathogenesis of a new SHIV in young RMs

We recently described a cohort of 13 therapy-naïve neonatal RMs that were SHIV-infected intravenously during 4–49 days post-birth and subsequently assessed for viral and antibody co-evolution up to 2 years post-infection (16). Two RMs were successfully infected with 5-ng p27 Ag of SHIV CH848 10.17 DT.E169K, and the remaining 11 RMs were infected following 50-ng p27 challenge (see Materials and Methods), but we found no association between the amount of virus used to establish infection and viral dynamics (Table 1) (16). SHIV CH848.10.17 DT.E169K is currently under evaluation in adult RMs, thus limiting data on viral pathogenesis when compared to other SHIVs that have been widely used in adult RMs (23 – 25). However, SHIV CH848 10.17 DT.E169K led to AIDS-related deaths 26–35 months post-infection in 4 of 13 animals (16) (Table S1), demonstrating that this SHIV was pathogenic.

**TABLE 1:** Cohort of SHIV-infected neonatal RMs^*a*^

Animal ID	Sex	Viremia status	Time of infection, post-birth	Virus inoculum (ng/p27)	*Mamu* alleles			First timepoint of viremia control
					A*01	B*08	B*17	
V035	Male	Controller	Day 49	5	−	−	+	Month 2
V059*	Female	Controller	Day 28	50	−	−	−	Month 8
V250	Male	Controller	Day 28	50	−	−	−	Month 14
V252	Female	Controller	Day 4	50	−	−	+	Month 12
V253*	Male	Controller	Day 4	50	−	−	+	Month 8
V055	Male	Non-controller	Day 49	50	−	−	+	n/a
V058	Male	Non-controller	Day 28	50	−	−	−	n/a
V093	Female	Non-controller	Day 42	50	−	−	−	n/a
V094	Male	Non-controller	Day 4	5	−	−	+	n/a
V095	Female	Non-controller	Day 21	50	−	−	-	n/a
V096	Male	Non-controller	Day 4	50	−	−	+	n/a
V249	Female	Non-controller	Day 28	50	−	−	+	n/a

^*a*^ We established SHIV CH848 10.17 DT.E169K infection in neonatal RMs using either 5 or 50 ng/p27 of virus inoculum from days 4 to 49 post-birth (see also Materials and Methods). We defined 1 week as 7 days. The *Mamu* genotypes tested for each animal were reported as positive (+) or negative (−) for *Mamu* A*01, B*08, and B*17 alleles. Each animal was categorized as viremia controller or non-controller based on our criteria for control at ≤1,000 vRNA copies/mL to undetectable plasma viremia for ≥2 consecutive timepoints (see also Table S1). *Partial controllers, viral load rebounded >1,000 copies/mL without intervention with anti-CD8 mAb treatment. The initial timepoint for viremia control in the relevant animals (viremia controllers) following SHIV infection is listed in months (see also Fig. 1); n/a, not applicable for viremia non-controllers.

**Fig 1:**
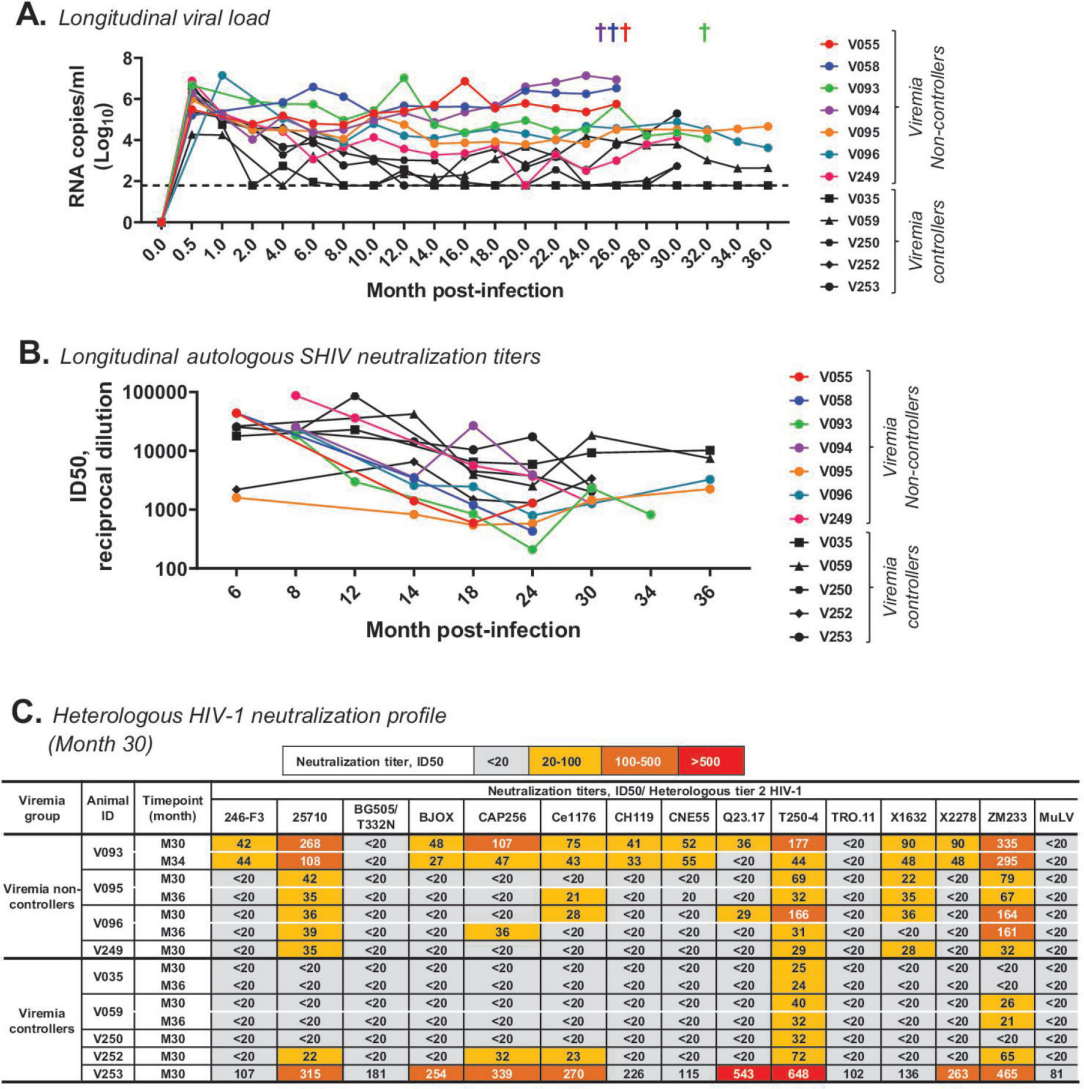
Pathogenesis of neonatal SHIV infection in RMs. (**A**) Dynamics of plasma viral load measured as SIV gag RNA via quantitative PCR and reported as RNA copies/mL (log10). Each symbol represents one animal; RMs with persistent high viral load of ≥~10^3^ RNA copies/mL were classified as viremia non-controllers and shown in colored circles, whereas macaques with viral loads of <10^3^ RNA copies/mL at two or more consecutive timepoints were classified as viremia controllers and shown in black symbols. The horizontal dash line represents the limit of detection of the assay (1.79 RNA copies/mL). The crosses (†) indicate the last timepoints tested for four animals (V055, V058, V093, and V094) that died of AIDS-related illnesses (see also Table S1); the crosses were color-coded to match the animal IDs. Animals 249, 252, and 253 were transferred to another program, so the available timepoints tested are shown. (**B**) Longitudinal plasma antibodies of up to 36 months following SHIV infection in RMs infected soon after birth were tested for neutralization of autologous SHIV CH848 10.17 dt.e169k in TZM-bl cells. Neutralization titer was reported as log ID50 (reciprocal dilution). Color codes and samples tested are described in panel A. (**C**) Heatmap summarizing the neutralization profile of plasma from month 30 post-SHIV infection in representative RMs (viremia controllers and non-controllers) that were tested against heterologous tier 2 HIV-1 strains in TZM-bl cells. Neutralization titer was reported as ID50. Plasma samples were tested for neutralization in TZM-bl cells and reported as reciprocal dilution (ID50). Plasma samples were tested for neutralization at a starting dilution of 1:20 and continued threefold. Positive neutralization was reported as ID50 3× >background (SVA-MuLV). Negative or background neutralization is shown in gray fill.

Following neonatal SHIV CH848.10.17 DT.E169K infection, a subset of young RMs (*N* = 5) demonstrated spontaneous control of viremia in blood (≤1,000 RNA copies/mL) at ≥2 consecutive timepoints in the absence of cART (Fig. 1A; Table 1; Table S1). Neonatal RMs were genotyped for major histocompatibility complex (MHC) class I alleles (*Mamu* A*01, B*08, and B*017), which have been shown to be associated with a dominant cytotoxic T-lymphocyte (CTL) response that controls disease progression in simian Immunodeficiency virus (SIV) and SHIV-infected rhesus macaques (26 – 29). Of 12 RMs studied, all were *Mamu*-A01 and B*08 negative, whereas three of five viremia controllers and four of seven viremia non-controllers were *Mamu*-B*17 positive (Table 1). Thus, these data suggested that *Mamu*-restricted CTL responses alone were not responsible for spontaneous control of viremia in the 12 SHIV CH848 10.17 DT.E169K-infected neonatal RMs in this study.

All 12 RMs studied generated autologous SHIV nAbs over time (Fig. 1B). Moreover, we found that at months 30–36 post-infection, four of four viremia non-controllers demonstrated evidence of neutralization against multiple (≥3) heterologous tier 2 HIV-1 strains, in contrast with two of five viremia controllers (Fig. 1C). Of three RMs (V093, V096, and V253) with the highest heterologous HIV-1 nAb titers (≥100 ID50), two animals (V093 and V096) were viremia non-controllers, whereas one animal (V253) was a viremia controller (Fig. 1C). Thus, here we described a pediatric animal model where HIV-1 nAbs, including heterologous HIV-1 nAbs characteristic of bnAbs, were generated independent of viremia control following 3 years of therapy-naïve SHIV-infection—this study has never been reported. These data also demonstrated that HIV-1 nAbs alone were not responsible for viremia control. We next robustly interrogated additional host immune parameters that potentially explained the immunologic milieu of viremia suppression following a pathogenic SHIV infection in young RMs.

### Profile of plasma analytes following neonatal SHIV infection

We interrogated plasma biomarkers associated with neonatal macaque SHIV infection by measuring levels of 48 non-human primate cytokines, chemokines, and growth factors using a multiplexed bead-based approach in the plasma previously collected from the 12 SHIV CH848 10.17 DT.E169K-infected RMs studied. Plasma from all RMs were assayed at three timepoints after SHIV infection: timepoint 1, 4 months post-infection (T1); timepoint 2, 6–8 months post-infection (T2); and timepoint 3, 10–14 months (T3). These timepoints overlapped with the onset of functional changes in host immunity, including plasma antibody profiles (16), and the dynamics of viremia in the SHIV-infected young RMs.

**Fig 2:**
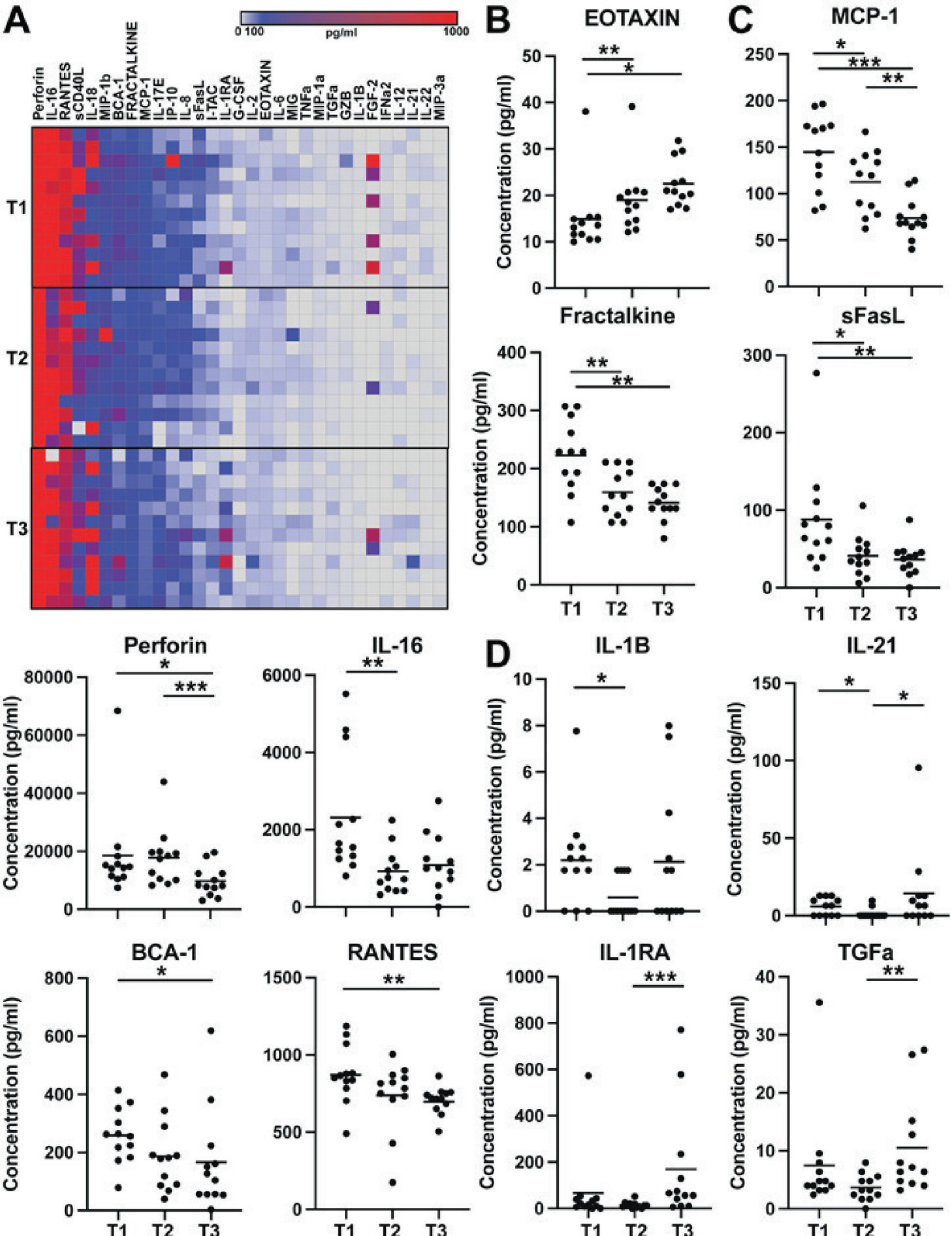
Peripheral blood cytokines and chemokines following neonatal SHIV infection in RMs. (**A**) Heatmap of concentrations (pg/mL) for the detectable analytes measured in the plasma from RMs at different timepoints. Analytes ordered in median concentration for all RMs. Timepoint 1, 4 months post-infection (T1); timepoint 2, 6–8 months post-infection (T2); and timepoint 3, 10–14 months post-infection (T3). Of 48 analytes, 31 were detectable: sCD40L, BCA-1, Eotaxin, FGF-2,IL-16, sFasL, G-CSF, IFN-α2, IL-2, IL-6, GZB, IL-8, IL-1RA, IL-1β, IL-12, IL-17E, IL-21, IL-18, IL-22, IP-10, MCP-1, I-TAC, MIG, MIP-1α, MIP-3α, MIP-1β, TNF-α, Rantes, TGFα, perforin, and Fractalkine (CX3CL1). Seventeen analytes were undetected: sCD137, GZA, GM-CSF, IFN-γ, IL-1α, IL-10, IL-15, IL-17A, IL-23, IL-28A, IL-31, IL-33, IL-4, IL-5, IL-7, VEGF-A, and TNF-β. (**B–D**) Plots showing concentration of plasma analytes for individual animals that had significant changes at any of the three timepoints studied (T1–T3). *P ≤ 0.05, **P ≤ 0.01, ***P ≤ 0.001; Wilcoxon rank-sum test, GraphPad Prism version 9.0.

Of 48 analytes interrogated, 31 were in the quantifiable range of the assay standards at any of the timepoints studied (Fig. 2A). The other 17 analytes could be present in the plasma but were below the quantifiable limit of detection of our assay. Perforin, interleukin (IL)-16, and Rantes (CCL5) were detected at the highest median levels (≥500 pg/mL), and soluble CD40 ligand (sCD40L), IL-18, MIP-1b (CCL4), BCA-1 (CXCL13), Fractalkine (CX3CL1), and MCP-1 (CCL2) were also highly detected (median ≥100 pg/mL) in the 12 animals across the three timepoints using this assay (Fig. 2A).

First, we determined analytes that significantly changed over time after SHIV infection, regardless of viral control status, and identified 12 analytes that significantly changed (*P* ≤ 0.05, Wilcoxon rank-sum test) between timepoints when compared pairwise (Fig. 2B through D). Eotaxin (CCL11) was the only analyte that was increased from T1 to T2 and T1 to T3 (Fig. 2B). There were seven other analytes (MCP-1, Fractalkine, sFasL, perforin, IL-16, BCA-1, and Rantes) that were either significantly changed between T1 and T2 and/or T1 and T3 that all were decreased at later timepoints compared to timepoint 1 (Fig. 2C). Four analytes (IL-1B, IL-21, IL-1RA, and TGFα) were decreased at T2, but then increased at T3 compared to T2 (Fig. 2D).

### Plasma analytes associated with viral suppression following neonatal SHIV infection

Next, we interrogated plasma analytes in representative RMs with viral suppression of ≤1,000 RNA copies/mL (controllers) in comparison with RMs where viral load persisted above 1,000 RNA copies/mL (non-controllers) over the course of 3 years of infection without cART. We studied the levels of plasma analytes in three representative viremia controllers (V035, V250, and V252) that had viremia control ranging from 62 (undetected) to 1,530 vRNA copies/mL, and we studied the levels of plasma analytes in six representative viremia non-controllers (V055, V058, V093, V094, V095, and V096) that had viral loads ranging from ~7,000–7,000,000 copies/mL for comparison (Table S1).

**Fig 3:**
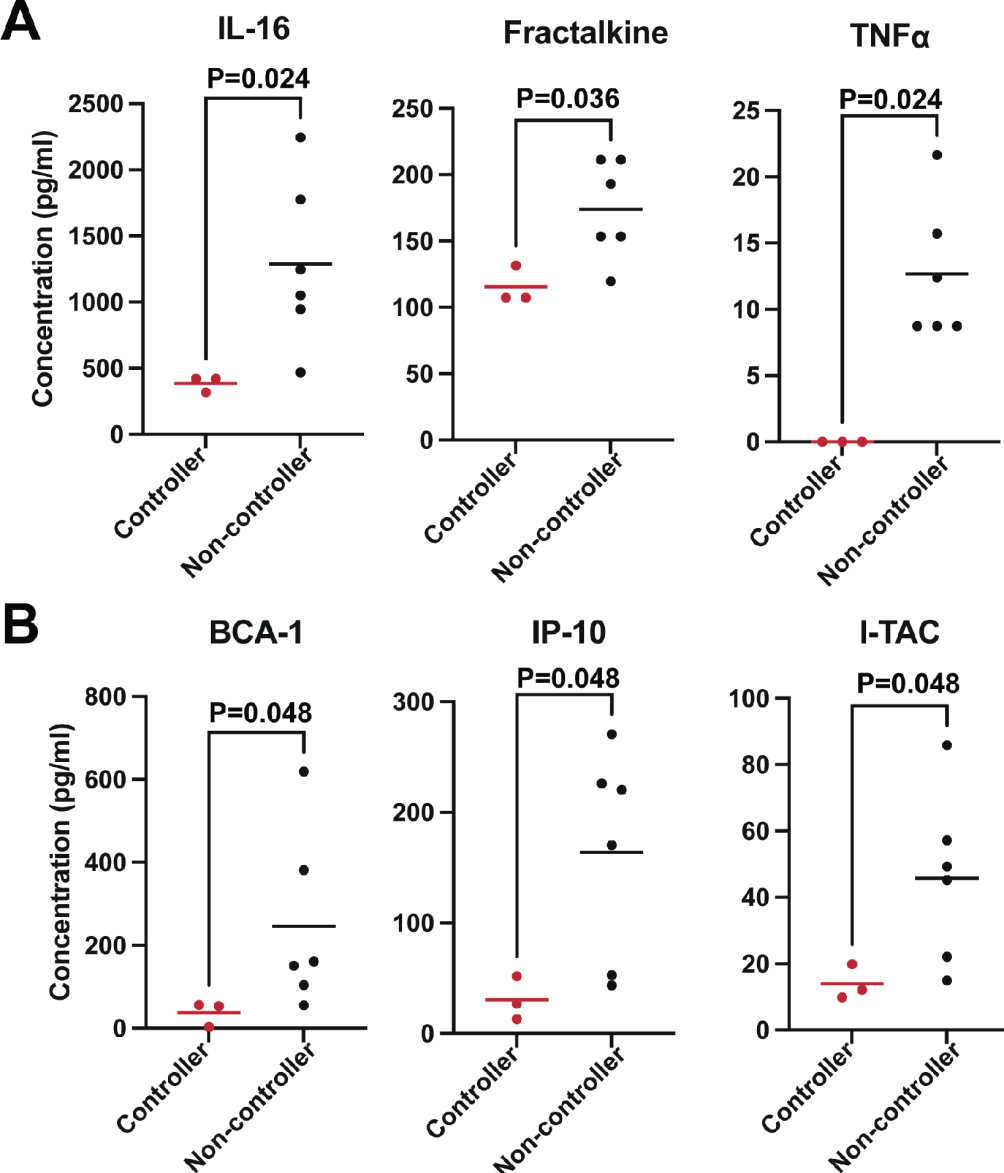
Plasma analytes in controllers and non-controllers of neonatal SHIV infection in RMs. (**A and B**) concentration of plasma analytes for individual RM that had viral control (control, *N* = 3) or persistent SHIV viremia (no control, *N* = 6) that were significantly different (*P* ≤ 0.05, Wilcoxon-Mann-Whitney) at timepoint 2 (6–8 months) (**A**) or timepoint 3 (10–14 months) (**B**).

There were no significant differences in levels of the 31 detected analytes (Fig. 2A) in viremia controllers compared to non-controllers during T1 (not shown). In contrast, we found that viremia controllers had significantly lower levels of IL-16 (*P* = 0.024), Fractalkine (*P* = 0.036), and TNF-α (*P* = 0.024) during T2 (Fig. 3A), and significantly lower levels of BCA-1 (*P* = 0.048), IP-10 (*P* = 0.048), and I-TAC (CXCL10) (*P* = 0.048) during T3 (Fig. 3B), compared to viremia non-controllers. BCA-1 has been shown to be associated with HIV germinal center responses (30). That BCA-1 was elevated in viremia non-controllers supports the notion that persistent antigen stimulation of B cells facilitates the induction of heterologous nAbs in these viremia non-controllers as previously described (16). These data also imply that heterologous nAbs alone may be ineffective for complete viral suppression. IP-10 and I-TAC have been shown to be correlated with HIV-1 severity in humans as monocytes and dendritic cells (DCs) produce IP-10 in response to HIV-1 infection and promote latent infection in resting memory CD4 T cells by facilitating HIV entry and integration (31 – 33). Moreover, I-TAC is a CXCR3-ligand that when elevated in the plasma has been shown to promote CD4 T-cell (FoxP3+) activation that was also associated with severe disease progression in HIV-1 infection (32, 34). Thus, these data implied that viral suppression following neonatal SHIV infection in RMs was associated with an immunologic milieu of less inflammatory cytokines that are linked with severe disease outcome or establishment of latent infection. This inflammatory milieu in viremia non-controllers also included evidence of activated germinal centers that supported nAb induction. For further understanding of the immunologic milieu defined by the plasma analyte profiling, we next interrogated host immune cells in viral suppression.

### Landscape of immune cells in viral suppression during early timepoints of neonatal SHIV infection

In total, we studied 81,314 total peripheral blood mononuclear cells (PBMCs) from six representative RMs at months 6–8 (early timepoints) and/or months 14–16 (later timepoints) post-infection: V035, V055, V059, V094, V252, and V253 (Table 2). We defined enriched cell subsets as having a statistically significant higher frequency across the groups using chi-squared test (R version 4.2.2).

**TABLE 2:** Representative SHIV-infected neonatal RMs for single-cell immune repertoire analyses^*a*^

Animal ID	Timepoint post-infection	Viremia status	Infection timepoint	RNA copies/mL	Number of cells recovered
V055	Month 6	Non-controller	Early	63263	15,999
V094	Month 6	Non-controller	Early	23228	10,942
V035	Month 8	Controller	Early	<62	16,133
V059*	Month 8	Controller	Early	<62	5,530
V252	Month 14	Controller	Late	<62	11,528
V253*	Month 14	Controller	Late	<62	8,870
V035	Month 16	Controller	Late	<62	12,312

^*a*^ From each of seven samples (six young RMs infected as neonates) listed in the table above (animal ID and timepoint), we studied ~20,000 PBMCs via 10× Genomics single-cell immune profiling assay (Pleasanton, CA). Controllers had ≤1,000 vRNA copies/mL to undetectable plasma viremia for ≥2 consecutive timepoints. The limit of detection was 62 RNA copies/ mL. We recovered a total of 81,314 cells for our analyses. *Partial controllers; viral load rebounded >1,000 copies/mL without intervention with anti-CD8 mAb treatment. We defined early infection timepoints as <12 months post-SHIV infection and late infection timepoints as >12 months post-SHIV infection.

**Fig 4:**
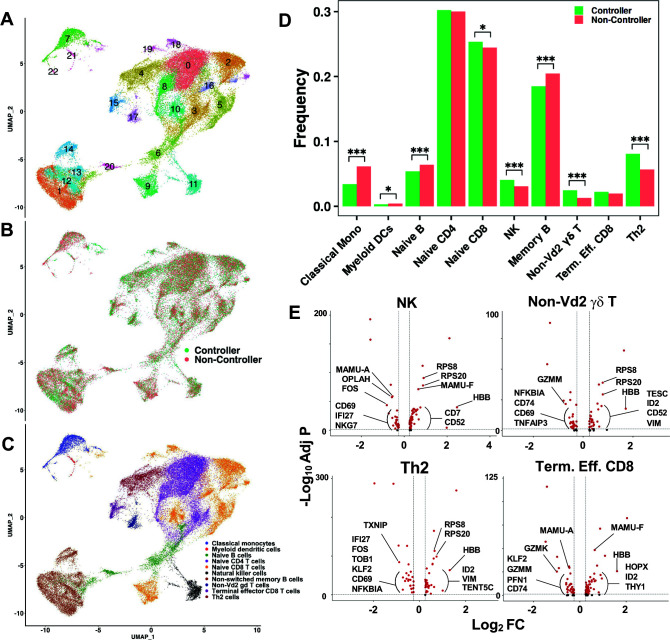
Immune cells in controller and non-controller RMs during early timepoints of SHIV infection. (**A**) Uniform Manifold Approximation and Projection (UMAP) plot showing 23 unique transcriptional clusters in SHIV non-viremic (v035, month 8; v059, month 8) and viremic (v055, month 6; v094, month 6) RMs. (**B**) UMAP plot showing overlay of cells from non-viremic (green, controller) vs viremic (red, non-controller) RMs. (**C**) Cell types as predicted by SingleR overlayed on a UMAP plot. (**D**) Bar chart comparing the frequency of cell types in viremia controllers (green) vs non-controllers (red). Statistics: *P < 0.05, ***P ≤ 0.0005, using the chi-squared test comparing frequency between groups. (**E**) Volcano plots showing genes upregulated (to the right in the plots) or downregulated (to the left in the plots) in cell types that are more frequent during early timepoints of viremia control vs non-control. Red dots: genes with a log2 fold change >0.25 and adjusted *P* value of <0.05. Labeled red dots: genes with highest log2 fold change and adjusted *P* value. Annotated genes that were differentially expressed with log fold change of ≥0.25 and statistically significant adjusted *P* value of <0.05 are indicated in panel E. The total list of differentially expressed genes, including the unannotated genes, is shown in Table S2.

First, we studied single-cell transcriptional profiling of PBMCs in two viremia non-controllers at month 6 (V055 and V094) compared with two viremia controllers at month 8 (V035 and V059). Total PBMCs from these two groups of RMs clustered transcriptionally into 23 unique clusters (Fig. 4A) that were not based solely on the dynamics of viremia (Fig. 4B), suggesting that viremia control was dependent on functions rather than frequencies of immune cell subsets. The 23 unique transcriptional clusters defined 10 immune cell types and cell subsets (Fig. 4C).

#### Transcriptome profiling in viral suppression

We found several immune cell subsets that were enriched in viremia controllers compared to non-controllers during early timepoints of SHIV infection; NK, non-Vd2 gamma-delta (γδ) T, T helper 2 (Th2), and naïve CD8 T cells (Fig. 4D). In NK, non-Vd2 γδ T and Th2 cells in viremia controllers, we found that ribosomal proteins *(*RPS8 and RPS20) were upregulated (Fig. 4E; Table S2), suggestive of these cell types being more transcriptionally active in viremia controllers. Previous studies have shown that transcriptional regulator ID2 mediates CD8 T-cell immunity by promoting differentiation and survival of cytotoxic T cells (35, 36). Interestingly, we found that ID2 was upregulated in T-cell subsets (non-Vd2 γδ T, Th2, and terminal effector CD8) in viremia controllers (Fig. 4E; Table S2). Terminal effector CD8 T cells were found at a higher frequency in viremia controllers compared to non-controllers, but the difference was not statistically significant (Fig. 4D). Thus, these data raised the hypothesis that viremia controllers had activated immune cells with anti-viral and cytotoxic activities, including NK and cytotoxic T cells of different lineages when compared to viremia non-controllers during early timepoints of neonatal SHIV infection.

#### Transcriptome profiling in viral persistence

In viremia non-controllers, we found that granzymes K (GZMK) and M were upregulated in terminal effector CD8 and non-Vd2 γδ T cells (Fig. 4E; Table S2). GZMK promotes T-cell exhaustion (37), thus suggesting that viremia non-controllers were enriched for exhausted terminal effector CD8 and non-Vd2 γδ T cells in contrast with viremia controllers (Fig. 4E; Table S2). Additionally, we found that viremia non-controllers had upregulated pro-inflammatory cytokines (NFKB, NKG7, IFI27, and TNFAIP3) and activation markers including CD69 (Fig. 4E; Table S2). That there was upregulation of CD69+ NK and T (non-Vd2 γδ T and Th2 cells) cells in viremia non-controllers suggested that these populations were dysfunctional. Moreover, viremia non-controllers upregulated markers that inhibited cell growth (TOB1) and promoted T-cell inactivation (KLF2). These data raised the hypothesis that viremia non-controllers had a high frequency of cytotoxic immune cells, including T cells with exhausted or dysfunctional phenotypes when compared to viremia controllers during acute neonatal SHIV infection.

### Landscape of immune cells in viral suppression during late timepoints of neonatal SHIV-infection

**Fig 5:**
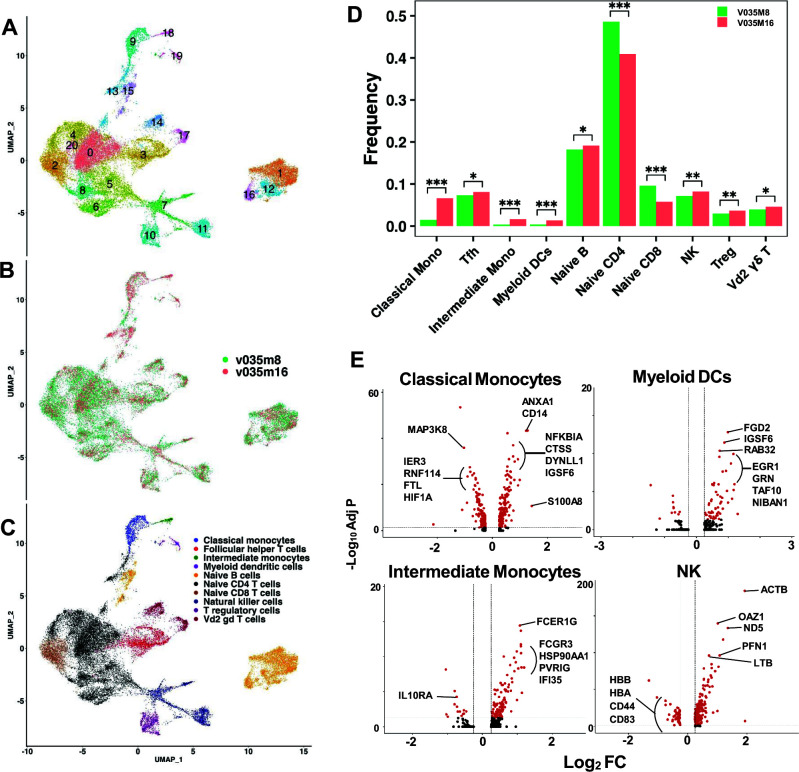
Immune cell subsets in viremia control during longitudinal early and late timepoints of SHIV infection. (**A**) UMAP plot showing 21 unique transcriptional clusters in longitudinal viremia control at months 8 and 16 of the same animal V035. (**B**) UMAP plot showing overlay of cells from v035, month 8 (green), vs v035, month 16 (red). (**C**) Cell types as predicted by SingleR overlayed on the UMAP plot shown in panel B. (**D**) Bar chart comparing the frequency of cell types in V035, month 8 (green), vs V035, month 16 (red). Statistics: *P < 0.05, **P ≤ 0.005, ***P ≤ 0.0005, using the chi-squared test comparing frequency between groups. (**E**) Volcano plots showing genes upregulated (right side of plots) or downregulated (left side of plots) in cell types that were more abundant at month 16. Annotated genes that were differentially expressed with log fold change of ≥0.25 and statistically significant adjusted *P* value of <0.05 are indicated in panel E. The total list of differentially expressed genes, including the unannotated genes, is given in Table S3.

**Fig 6:**
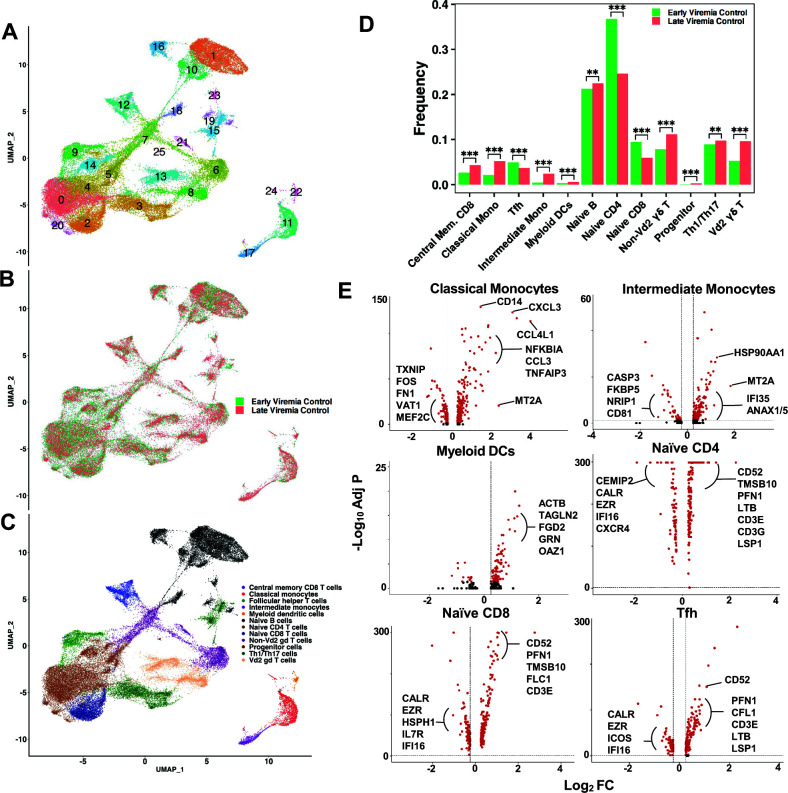
Immune cell subsets associated with viremia control during cross-sectional early and late timepoints of SHIV infection. (**A**) UMAP plot showing 26 unique transcriptional clusters in early (V035, month 8, and V059, month 8) vs late (V035, month 16; V252, month 14; and V253, month 14) timepoints of viremia control. (**B**) UMAP plot showing overlay of cells from early viremia control (green) vs late viremia control (red). (**C**) Cell types as predicted by SingleR overlayed on a UMAP plot shown in panel B. (**D**) Bar chart comparing the frequency of cell types in early viremia control (green) vs late viremia control (red). Statistics: **P ≤ 0.005, ***P ≤ 0.0005, using chi-squared test comparing frequency between groups. (**E**) Volcano plots showing genes upregulated or downregulated in representative immune cell types that are more abundant during early viremia control (naïve CD4, naïve CD8, and Tfh cells) or late viremia control (classical and intermediate monocytes, and myeloid dendritic cells). Directionality: right side of plots, genes upregulated in immune cell subset studied; left side of plots, genes downregulated in immune cell subsets studied. Annotated genes that were differentially expressed with log fold change of ≥0.25 and statistically significant adjusted *P* value of <0.05 are indicated in panel E. The total list of differentially expressed genes, including the unannotated genes, is shown in Table S4.

Second, we interrogated host cell immunity during early vs late timepoints of viremia control both longitudinally (within the same animal) (Fig. 5; Table S3) and cross-sectionally (across animals) (Fig. 6; Table S4).

#### Longitudinal viremia control in a single RM V035

Total PBMCs from early (M8) and late (M16) timepoints within animal V035 clustered transcriptionally into 21 unique clusters (Fig. 5A) that were not based solely on timepoint (Fig. 5B) and defined 10 immune cell types and subsets (Fig. 5C). We found that NK cells that were enriched during early timepoints of viremia control when compared with non-control (Fig. 4D) were also among the enriched immune cells during late timepoints of viremia control at month 16 when compared with month 8 in the same animal, V035 (Fig. 5D). NK cells during early control of viremia had upregulation of CD44 and CD83 that are associated with enhanced cytotoxicity (38), and activation and maturation of immune cells (39), respectively (Fig. 5E). NK cells during late control of viremia maintained an activated state as demonstrated by upregulation of lymphotoxin beta that is upregulated on activated lymphocytes, including NK cells (40) (Fig. 5E). These data suggested that activated NK cells are associated with viremia control during early and late timepoints of viremia control. Additionally, circulating CD4+ follicular helper (Tfh-like) and regulatory (Treg-like), and Vd2 γδ, T cells were enriched during late viremia control at month 16 in V035 compared to M8 (Fig. 5D). Terminal effector CD8, non-Vd2 γδ T, and Th2 cells that were enriched or elevated during early timepoints of viremia control when compared to viremia non-control (Fig. 4D), were not identified as immune cell subsets of viremia control at month 16 in V035 (Fig. 5D). These data implicated distinct T-cell subsets with functional roles at different stages of viremia control, but we cannot rule out limitations of SingleR classification for defining macaque immune T-cell subsets in different heterogeneous cell subsets analyzed.

In addition, enriched immune cell subsets detected at month 16 compared to month 8 included classical and intermediate monocytes and myeloid DCs (Fig. 5D). At month 16, classical monocytes had CD14 gene upregulation (Fig. 5E; Table S3), which is a monocyte-differentiating antigen that promotes NFKB activation, cytokine secretion, and inflammatory responses (41). Moreover, both *CD14* and *NFKB1A* gene upregulation in classical monocytes were suggestive of activated NFKB signaling and toll-like receptor signaling pathways (KEGG Pathways, STRING, version 11.2). Intermediate monocytes had upregulated *FCERIG* and *FCGR3* genes that encode immunoglobulin gamma Fc receptor precursors with various roles in cell-mediated cytotoxicity (42), in addition to upregulation of PVRIG that is an NK cell immune checkpoint inhibitor (43) (Fig. 5E; Table S3). PVRIG belongs to the family of inhibitory receptors and is also expressed on CD8 T cells, but inhibition of PVRIG has been shown to promote anti-tumor immunity of NK cells in mice and humans (43). Thus, intermediate monocytes with PVRIG upregulation represent a population of innate cells that remains to be fully characterized, and were associated with viremia control in young SHIV-infected RMs. Additionally, these data suggested a role for activated classical and intermediate monocytes in late viremia control at month 16 compared to early viremia control at month 8 in RM V035. That classical monocytes and myeloid DCs were also enriched in viremia non-controllers compared to controllers during early timepoints of neonatal SHIV infection (Fig. 4D) suggested that classical monocytes and myeloid DCs work in synergy with other cell subsets found in the immunologic milieu following SHIV infection to impact viremia control.

#### Cross-sectional viremia control during late timepoints of infection in three RMs

Following our observation for monocytes and DCs being enriched in late timepoints of viremia control in longitudinal SHIV infection in a single RM V035, we investigated the immune cell subsets in early (*N* = 2: V035 M8 and V059 M8) vs late (*N* = 3: V252 M14, V253 M14, and V035 M16) timepoints of SHIV infection in cross-sectional samples (Fig. 6). Total PBMCs from all five samples clustered transcriptionally into 26 unique clusters (Fig. 6A) that were not based solely on timepoint (Fig. 6B) and defined 12 immune cell types and subsets (Fig. 6C). Interestingly, monocytes and DCs were enriched in late viremia control (Fig. 6D), in agreement with viremia control during late vs early SHIV infection in a single animal (Fig. 5D). In addition to progenitor cells, multiple T-cell subsets were enriched in late vs early viremia in cross-sectional samples, including central memory CD8 T, non-Vd2 γδ T, Vd2 γδ T, and Th1/Th17 cells (Fig. 6D). Similarly, T-cell subsets were also enriched in viremia controllers vs non-controllers during early SHIV-infection (Fig. 4D). Thus, these data implicated an immunologic milieu of different T-cell subsets that may contribute to viremia control at different stages of macaque SHIV infection in a pediatric model and not only canonical CD8 T cells that have been extensively studied for their roles in spontaneous viremia suppression.

Additionally, we found naïve CD4 and CD8 T cells as well as circulating Tfh-like cells at lower frequencies compared to other cell types during late timepoints for viremia control compared to early timepoints of viremia control in cross-sectional samples (Fig. 6D), which is consistent with differentiation and maturation of these cells presumably by viral antigens during the course of infection. For example, central memory CD8 T cells were enriched in late viremia, while naïve CD8 T cells were significantly decreased (Fig. 6D). However, we cannot rule out that decreased frequencies of naïve cell subsets over time were due to cell death (Fig. 5D and 6D).

### Dynamics of CD8-expressing cells during neonatal SHIV infection

**Fig 7:**
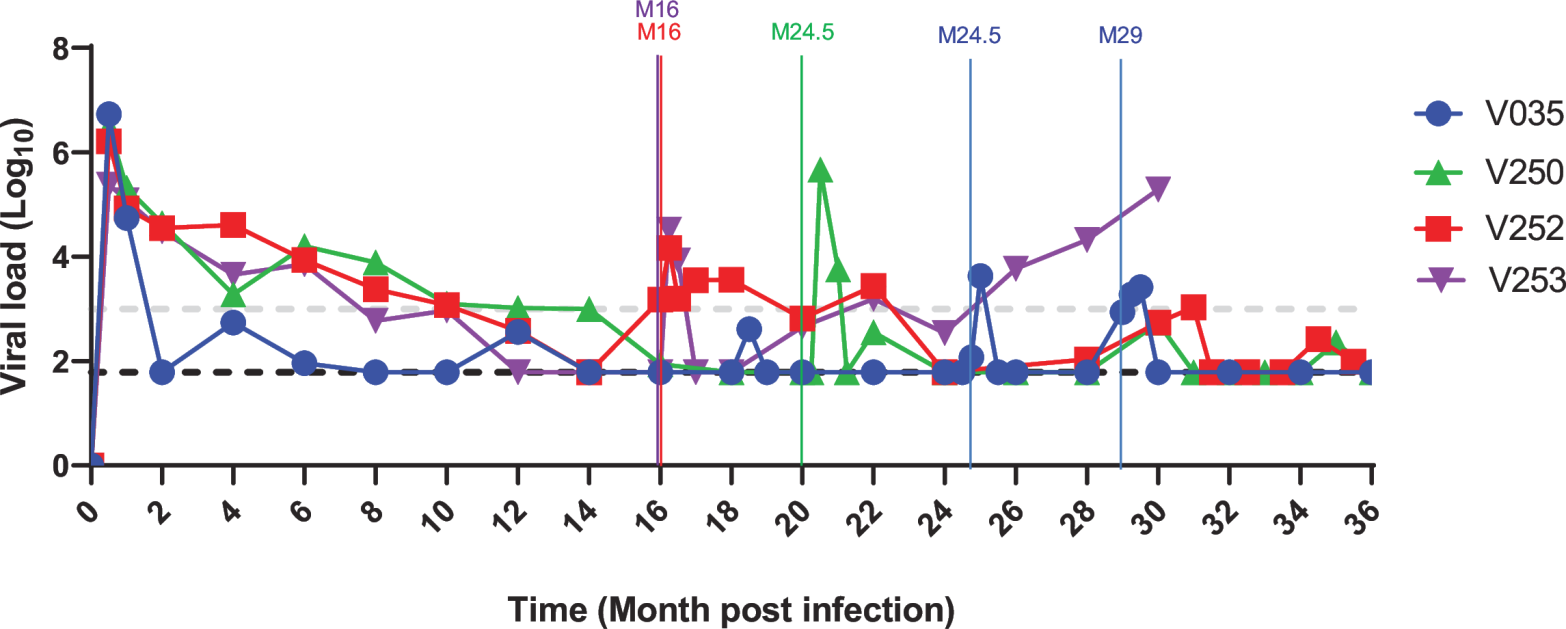
Viral dynamics in viremia controllers, (±) anti-CD8β-depleting antibody treatment, following neonatal SHIV infection. The gray dashed line indicates the cutoff for defining viremia control at 1,000 RNA copies/mL, and the black dashed line indicates the limit of detection of 62 RNA copies/mL. Shown are plasma viral RNA copies in four RMs (V035, V250, V252, and V253) with viremia control during neonatal SHIV infection. The vertical lines indicate the timepoints of intravenous infusion of 50 mg/kg for anti-CD8β monoclonal antibody CD8β2255R1 per animal; the color of the line is an indication of the animal that was treated at that timepoint.

CD8-expressing cells play key roles in controlling viremia during human HIV and macaque SHIV infection (18, 44, 45). Transcriptomics of PBMCs in neonatal SHIV infection revealed a potential role for CD8-expressing cells including CD8 T and NK cells during early (Fig. 4D) and late (Fig. 6D) viremia control. In four representative viremia controllers (V035, V250, V252, and V253), we tested whether infusions of anti-CD8β depleting antibody CD8β2255R1 would alter viral dynamics as previously described (46, 47), presumably via transient inhibition of CD8β-expressing cells that constitute the dominant proportion of CD8 T cells (48). We observed viral rebound or increase in viral load in all four viremia controllers (Fig. 7; Table S5). Viral load increase following anti-CD8β treatment occurred within 1 week of antibody treatment, but within 2–3 weeks, the viral load was restored to similar levels found prior to antibody treatment. We hypothesized that viral rebound was due to transient inhibition of CD8β-expressing T cells as described (47), in agreement with our findings that anti-CD8β treatment did not prevent CD8 T-cell generation at later timepoints following antibody treatment (Table S5). Moreover, we found that both viremia controllers and non-controllers generated similar absolute levels of CD4 and CD8 T cells at select timepoints tested based on sample availabilities (Table S5), thus supporting a role for the frequencies of different functional T-cell subsets in modulating viremia suppression during neonatal SHIV infection.

### Macaque TCR and B-cell receptor repertoires in neonatal SHIV infection

**Fig 8:**
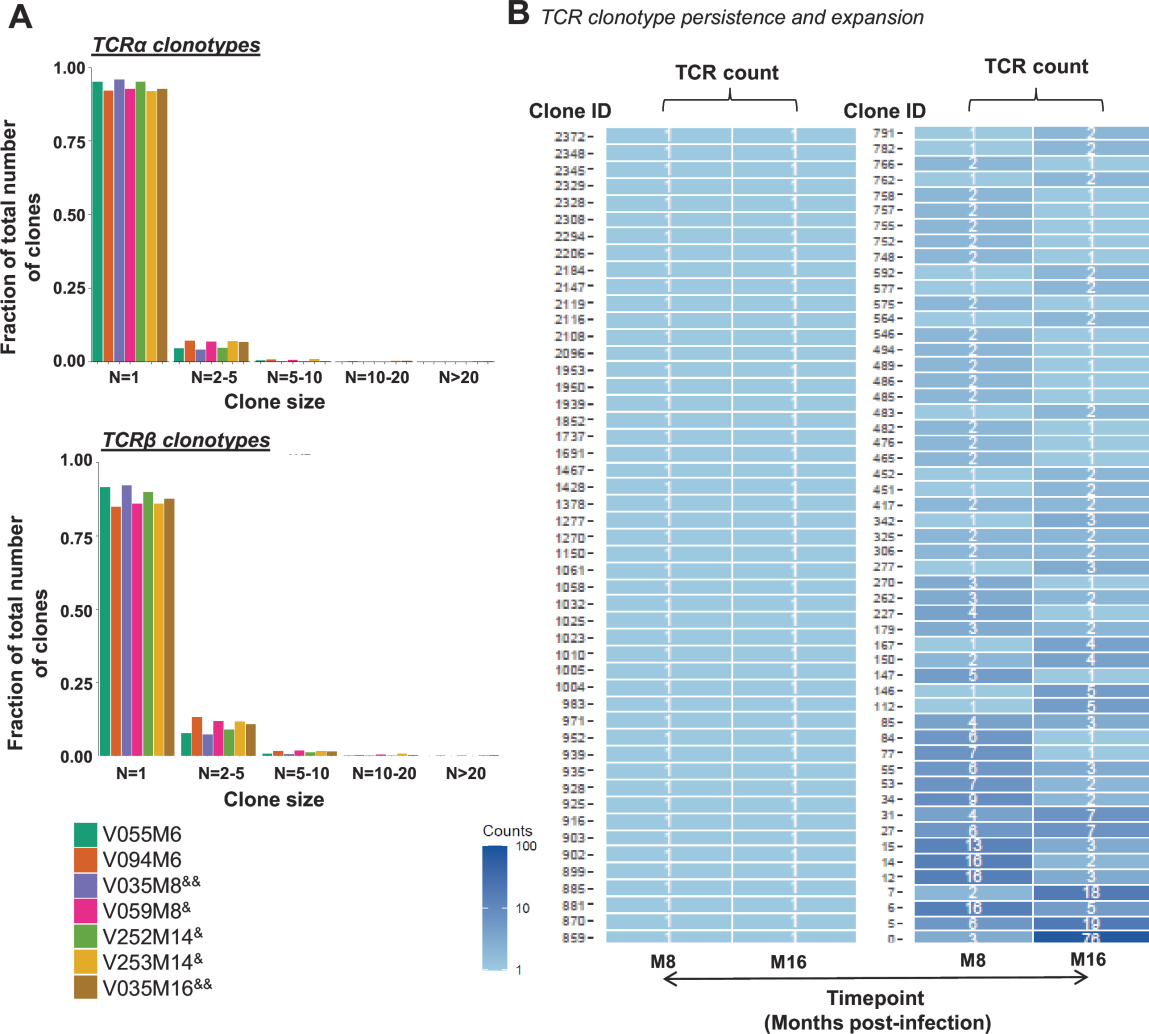
Macaque TCR sequencing. (**A**) Size of unique T-cell clones from PBMCs in RMs at different timepoints following neonatal SHIV infection. Sample ID – animal ID and timepoint post-infection; controllers (^&^) and controller with longitudinal sample (^&&^), whereas non-controllers had no symbols. Data shown are TCR clonotypes of various sizes or cell counts, and the fraction of each T-cell clonotype within the total normalized clones studied. T-cell clone sizes based on number of members per clonotype were grouped as follows; *N* = 1, 2–5, 5–10, 10–20, and >20. Clonality was determined based on T-cell receptor alpha (α) (top panel) and beta (β) (bottom panel) sequencing. (**B**) Dynamics of TCR clones in PBMCs from longitudinal timepoints (month 8 vs month 16) of infection in a single RM (V035). Clonality was based on TCRβ sequencing. Clone size is shown by the key. TCR clonotypes are based on identical nucleotides in the TCRβ variable gene.

Adaptive immunity that involves antigen-specific responses is regulated by TCRs and B-cell receptors (BCRs) on T and B cells, respectively (49). Moreover, clonal expansion of B/T cells is a hallmark of antigen selection in diseases that may be captured via TCR and BCR sequencing (50 – 52). In addition to generating transcriptome sequences of single cells in PBMCs from young RMs infected as neonates using the single-cell immune profiling assay (10× Genomics) (Fig. 4 to 6), we sequenced paired genes in the BCRs and TCRs. We sequenced paired TCRα and TCRβ chains from circulating single T cells in PBMCs collected from RMs at different timepoints following neonatal SHIV infection (Fig. 8A; Table 2). Interestingly, in one representative RM V035 with most effective viremia control (longitudinal viral loads and length of control), we found TCR clonotypes of identical nucleotides in the TCRβ variable gene, often used to define TCR clonality (53), at M8 post-infection (~6 months old) that persisted and/or expanded in M16 (~14 months old) (Fig. 8B). Thus, these data demonstrated T-cell clonal expansion following neonatal SHIV infection in RMs that may contribute to viremia control, in agreement with transcriptomic analysis of immune cell subsets in viremia control post-SHIV infection (Fig. 5D and 6D).

**Fig 9:**
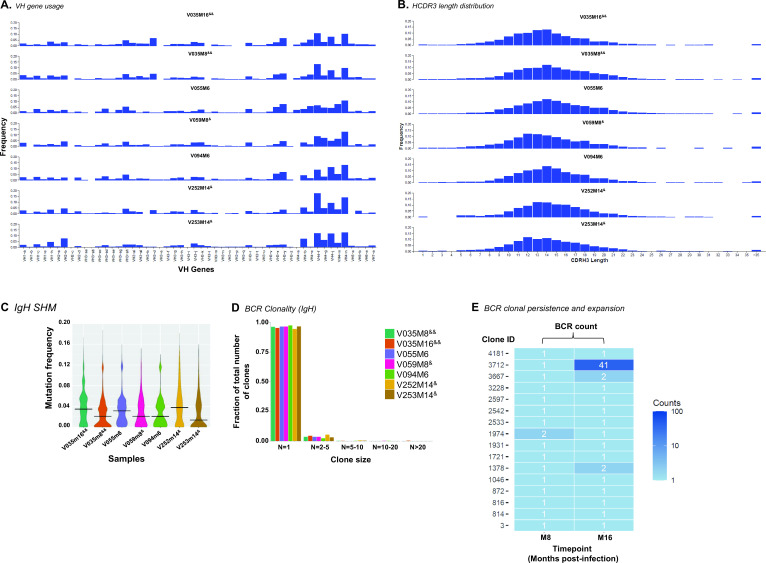
Macaque BCR repertoires following neonatal SHIV infection. Paired heavy (VH) and light (VL) chain genes or BCRs from B cells in PBMCs that were studied using 5′ VDJ and GEX sequencing (10× Genomics). We recovered paired VH + VL genes from 7906 single B cells. The numbers of B cells for which we recovered VH genes from each animal were as follows: V035 M16, 1017; V035 M8, 1604; V055 M6, 2012; V059 M8, 629; V094 M6, 1080; V252 M14, 1005; and V253 M14, 559. (**A**) Shown is the frequency of VH gene usage normalized by clones for macaque VH genes from the B cells in each sample. The macaque VH gene usage was determined using Cloanalyst software containing macaque immunoglobulin database. (**B**) HCDR3 length distributions of VH genes used by BCRs recovered from each RM. Shown are the frequencies of BCRs with HCDR3 lengths ranging from 1 to >35 amino acids; the data shown were normalized by clones. (**C**) Violin plot summarizing the estimated SHM frequencies in the VH genes of BCRs studied per sample. The horizontal bar represents the median. (**D**) Data shown are clones of various sizes or cell count and the fraction of each B-cell clone within the total normalized clones studied. B-cell clone sizes based on the number of members per clone were grouped as follows; *N* = 1, 2–5, 5–10, 10–20, and >20. BCR clonality based on similar VD_H_J_H_ rearrangement. (**E**) Dynamics of BCR clones in PBMCs from longitudinal timepoints (month 8 vs month 16) of infection in a single RM (V035). Clone size is shown by the key. Clonality was based on similar VD_H_J_H_ rearrangements, >80% nucleotide identity in the HCDR3, and identical HCDR3 length of functional VH genes.

Additionally, we recovered 7,906 BCRs from single B cells in PBMCs of all seven samples isolated from six RMs following neonatal SHIV-infection. Fig. 9A showed that all samples used varied VH genes, but macaque VH1, VH3, and VH4 were the most commonly used. The length of the complementarity determining region 3 (CDR3) of the VH gene and somatic hypermutation (SHM) frequencies are genetic properties associated with antibody functions, especially for HIV-1 envelope-reactive antibodies with effective neutralization capacity (54 – 56). The BCRs in all samples used a wide range of HCDR3 lengths (Fig. 9B) and estimated median nucleotide SHM frequencies up to 4% (Fig. 9C). Like the TCR clonotypes (Fig. 8A and B), the majority of the BCR clones in SHIV-infected neonatal RMs had a single lineage member (Fig. 9D), but animal V035 with longitudinal viremia control had clonal lineages at month 8 that persisted at M16 (Fig. 9E). Clonally related B cells that persisted in V035 from M8 to M16 were IgA/ IgD/ IgE/ IgM isotypes with SHMs in the heavy and light chain genes (not shown), and the BCRs of these B cells will be further evaluated as recombinant monoclonal antibodies to test their anti-viral properties. Naïve B cells were enriched in late viremia control compared with early control in longitudinal V035 samples (M8 vs M16) (Fig. 5D) as well as cross-sectional analysis of different samples representing early vs late control (Fig. 6D). That we did not detect clonal persistence of mutated IgG BCRs in V035 at M16 post-infection implicated subdominant antigen-driven clonal expansion of class-switched B cells that were not detected via transcriptomics.

## DISCUSSION

Among HIV-1-infected individuals are rare elite controllers, representing only <1% of adult and pediatric HIV-1-positive cases, who spontaneously suppress viremia to undetectable viral loads while maintaining elevated CD4 T-cell counts without receiving anti-retroviral therapy (57, 58). Thus, the mechanisms underlying elite control have been aggressively sought to guide the design of new vaccines and therapeutic strategies (59). In this study, we defined biomarkers associated with viremia suppression following neonatal SHIV infection in RMs as low levels of pro-inflammatory cytokines in an immunologic milieu of enhanced activated immune cell subsets, including cytotoxic CD8 T and NK cells, as well as innate cells such as DCs and monocytes. Moreover, evidence of TCR clonal persistence and expansion over time in SHIV-infected neonatal RMs with viremia suppression implicated a role for antigen-specific T cells in mediating viremia control in young RMs following neonatal SHIV infection. Using robust approaches, we studied a new pediatric model of SHIV infection that elicited heterologous HIV-1 nAbs independent of viremia control and found new candidate immune cell subsets in addition to previously described cytotoxic CD8 T and NK cells that were associated with spontaneous viremia suppression.

Elite control may be a temporary state; thus, it is important to be able to distinguish and further characterize elite controllers with long-term clinical success from those with disease progression to AIDS (59). In general, HIV-1-infected children progress faster to AIDS than adults, and there is also a higher frequency of viremic long-term non-progressors that do not develop AIDS among pediatric HIV-1 infections (58). One limitation of our study was that we did not measure CD4 T-cell counts throughout neonatal SHIV infection; thus, it is unknown whether any of these animals would be classified as elite controllers. However, of the four viremia controllers (V035, V250, V252, and V253), none died from AIDS-related complications up to 3 years of SHIV infection without receiving anti-retroviral therapy, consistent with pediatric non-progressors (17, 60).

Robust HIV-1-specific CD4 and CD8 T cells were recently found to be associated with viremia control in pediatric subjects with at least 1 year of undetectable plasma viremia (17). In this study, single-cell transcriptomics of peripheral blood cells from SHIV-infected neonatal RMs suggested that viremia suppression was associated with a higher frequency of activated immune cells including T cells, NK cells, and intermediate monocytes with cytotoxic functions as defined by their enriched gene expression profiles. Whether we compared viremia and non-viremia control during early SHIV infection or viremia control in early vs late SHIV infection, T-cell subsets including CD8 memory, Treg, and γδ were among the most predominant cell types associated with viral suppression. Additionally, we observed viral rebound of one to four logs following anti-CD8β depleting antibody treatment in four representative viremia controllers (V035, V250, V252, and V253), and TCR sequencing data were suggestive of clonal expansion over time in one representative viremia controller (V035). Thus, these data were in agreement with T cells playing a key role in viremia control following neonatal SHIV infection as described for human pediatric elite controllers (17).

Previous studies described NK cells and DCs as the innate immune cell subsets associated with spontaneous control of HIV-1 infection (21), in agreement with our findings (Fig. 5 and 6). Additionally, we identified activated intermediate monocytes with upregulation of the gene expressing the inhibitory receptor PVRIG, which has been shown to regulate NK cell-mediated anti-tumor activity (43). That activated monocytes with PVRIG upregulation were enriched during late vs early viremia implied that they play a role in inhibiting cytotoxic immune cells when not activated. Monocytes differentiate into macrophages and DCs; thus, it is of interest to know how PVRIG upregulation in monocytes of young SHIV-infected RMs impacts the monocyte differentiation process and functions of the differentiated cells. Moreover, spleen monocytes have been reported to mediate terminal differentiation of peripheral NK cells with effector functions using an *in vivo* model of tumor immunity (61). It is of interest to know whether NK cell differentiation and maturation would be facilitated or hindered by monocytes with PVRIG upregulation in the context of human HIV or macaque SHIV infection.

We observed that an increased number of plasma analytes were expressed at higher levels during early infection (T1), many of which were described in migration or recruitment (IL-16, IL-1 β, MCP-1, CX3CL1, and sFasL), differentiation (BCA-1 and IL-21), proliferation (CX3CL1), and activation (IL-21) of immune cells, including lymphocytes such as B cells, T cells and monocytes. BCA-1 has been implicated in HIV germinal center responses (30), and IL-21 plays a critical role in T cell-dependent B-cell activation, germinal center reactions, and humoral immunity (62), as well as CD8 T-cell activation (63). Thus, our data implicated an activated adaptive cellular response in early neonatal SHIV infection. Additionally, increased levels of sFasL that is involved in neutrophil-mediated apoptosis of T cells (64), and perforin that is released by killer cells during cytotoxic responses (65), that were detected during early infection implicated early cytotoxic activity by host immunity in neonatal SHIV infection that may contribute to control of viremia.

Interestingly, analytes that were detected at significantly higher levels only at later timepoints were Eotaxin, a potent chemoattractant (66, 67); IL-1RA, an IL-1 receptor antagonist (68); and TGFα, a potent immunosuppressive factor (69). CCR3, which is a receptor for Eotaxin, is also expressed on Th2 lymphocytes (67). Rantes attracts immune cells to sites of inflammation and also serves as an antagonist to Eotaxin. That Eotaxin increased in plasma concentration over time, while Rantes decreased over time suggested more activation of Th2 lymphocytes to provide B-cell help for antibody production or activation of eosinophils to support an allergic inflammatory environment (67). Additionally, IL-21 was expressed at higher levels in plasma at T3 vs T2. Thus, these data are suggestive of activated B and T cells following neonatal SHIV infection that persisted over time.

Viral persistence in neonatal SHIV infection compared to viremia suppression was associated with higher plasma levels of pro-inflammatory cytokines that may support chronic immune activation and ultimately immune cell dysfunction and senescence (13, 70) in agreement with a hypothesis generated from the single-cell transcriptomics data. For example, we found that peripheral blood cells in RMs with viral persistence had enriched genes including granzymes that are proteases that serve as mediators of cell death, indicative of cells with more dysfunctional state (37, 71). Thus, here we show the cross talk between plasma cytokine profiling and circulating immune cell transcriptomics to delineate host immunity of viremia control following neonatal SHIV infection.

Previous studies have shown that HIV-1 Env-reactive antibodies persist in the absence of viremia for individuals on anti-retroviral therapy, but the B-cell subsets were not fully defined (72, 73). Interestingly, one viremia controller (V253) generated plasma heterologous HIV-1 nAbs during late timepoints of SHIV infection, thus raising the hypothesis of neutralizing B-cell maturation in the absence of circulating viremia, which could be mimicked by cART. One possibility for B-cell maturation in the absence of circulating viremia is due to B-cell development in the presence of antigen in tissues such as lymph node-derived germinal centers, but our study was limited to circulating immune cell subsets. In this study, naïve B cells were enriched in viremia non-controllers compared with controllers during early timepoints of SHIV infection and were also enriched in late vs early viremia control in longitudinal and cross-sectional studies. That we detected clonally related mutated non-IgG VH genes in V035 at M8 and M16 suggested that class-switched IgG memory B cells were subdominant and may have been a consequence of B-cell dysfunction due to HIV infection as described (74). Other antibody isotypes such as IgA or IgM memory B-cell subsets have been reported to have anti-HIV functions (22, 75, 76); thus, the anti-viral roles of mutated IgA and IgM BCRs detected in V035 and isolation of heterologous HIV-1 nAbs from V253 are ongoing.

A limitation of our study was that the interpretations of the transcriptome data were based on previously annotated and classified genes, a common challenge in the area of transcriptomic analyses of host immunity. Thus, future studies will test the hypotheses generated by the single-cell transcriptomic data to improve our understanding of host immunity during viremia suppression in HIV-1-infected populations that may provide insights into new therapeutic strategies to achieve a functional cure (15). Overall, these data contributed to an understudied area of HIV-1 pathogenesis in pediatric subjects who constitute 15% of HIV-1 related deaths and 11% of new HIV-1 infections according to the World Health Organization’s 2021 report for the global HIV epidemic.

## MATERIALS AND METHODS

### SHIV design and viral stock production

CH848 10.17 N133DN138T (DT).E169K *env* encoded mutations that improve its propensity to bind BCRs targeting the V2-apex and V3-glycan bnAb epitopes as described (16). SHIV CH848 10.17 DT.E169K was generated in 293T cells, and virus concentration was estimated by p27 antigen (p27Ag) ELISA (Zeptometrix) as previously described (23). Animals were challenged with 5- or 50-ng p27Ag of SHIV CH848 10.17 DT.E169K intravenously in a final volume of 500 mL at BIOQUAL, Inc., Rockville, MD. We established infection in two animals with 5-ng p27Ag SHIV as initially planned: V035 and V094. However, some animals were initially challenged with 5-ng p27 SHIV but failed to establish infection, thus were subsequently re-challenged with 50-ng p27SHIV to achieve infection: V055, V058, V059, V093, and V095. Based on these initial observations, animals that were enrolled later in the study were challenged with only 50-ng p27 to establish infection: V096, V249, V250, V252, and V253. We found no association between the amount of virus used to establish infection and viral dynamics (set point and viral load) and immune outcomes such as binding and neutralizing antibody qualities and potencies (not shown).

### Animal cohort details

Our cohort of RMs were previously described, and all the animals were negative for the MHC class I allele (Mamu-A*01) (16). Note that viremia suppression or persistence was independent of the sex of the animals.

### Depletion of CD8β-expressing T cells

Anti-CD8β CD8β255R1 was provided by the Nonhuman Primate Reagent Resource. Concentration was 10.1 mg/mL, and it was dosed at 50 mg/kg. Dose route was subcutaneous shot between the shoulder blades. Antibody was provided in ready-to-use formulation. The site of the animal to be treated was clipped and prepped with alcohol. The ready-to-use material was drawn into appropriately sized sterile syringes and dosed. Dosing was recorded in electronic data capture system along with study records.

### Quantification of CD4 and CD8 T cells

Absolute CD4+ T-cell measurements were performed by using the BD Biosciences TruCount Platform (BD Biosciences, San Jose, CA) as described (77). This method was used to quantify absolute counts and percentages of subpopulations of CD3+CD4+ T cells, CD3+CD8+ T cells, and total CD45+ leukocytes. We reported the data for absolute counts because they were the exact number of CD4 or CD8 T cells present in the preset volume of whole blood analyzed by the commercially available assay (BD Biosciences TruCount) (Table S5). For fluorescence-activated cell sorter analysis, a minimum of 5,000 CD45+ leukocytes were acquired. For each run, as controls, we used Multicheck low-positive-control (stabilized human blood) samples; these yielded results consistent with expected values.

### Viral load measurement

Viral load was quantified as SIV RNA copies per milliliter as determined by a laboratory-developed two-step real-time quantitative PCR assay as described (16). The limit of quantification (LOQ) for this assay was determined by evaluating the exact binomial confidence boundaries and percent detection of the target RNA concentration of the lowest detected member of a validation panel. The LOQ for this assay is approximately 62 RNA copies per milliliter (1.79 log) of sample.

### Plasma analyte cytokine/chemokine/growth factor multiplex measurement

Levels of 48 cytokine, chemokine, and growth factors were determined using the bead-based multiplex assay based on the Luminex xMAP technology using the Milliplex non-human primate cytokine/chemokine/growth factor panel A 48-plex reagent kit (Millipore, #PRCYTA-40K-PX48) following the manufacturer’s standard protocol. The assay was performed using 25 µL of plasma from each animal at three timepoints (T1, T2, and T3) in technical duplicate that is incubated, premixed, fluorescently labeled magnetic microsphere beads with the 48 analytes (BCA-1, sCD137, sCD40L, Eotaxin, sFASL, FGF-2, Fractalkine, G-CSF, GM-CSF, granzyme A, granzyme B, IFN-α2, IFN-γ, IL-1α, IL-1β, IL-1RA, IL-2, IL-4, IL-5, IL-6, IL-7, IL-8, IL-10, IL-12 (p70), IL-15, IL-16, IL-17A, IL-17E, IL-18, IL-21, IL-22, IL-23, IL-28A, IL-31, IL-33, IP-10, I-TAC, MCP-1, MIG, MIP-1α, MIP-1β, MIP-3α, perforin, Rantes, TGFα, TNF-α, TNF-β, and VEGF-A. Data were acquired with the Luminex analyzer (MAGPIX) and Luminex xPONENT acquisition software. Median fluorescent intensity (MFI) is captured for all samples, and background was subtracted using a negative control well that included unlabeled beads and plasma (net MFI). Each analyte has seven concentration standards that are then utilized to generate a standard curve and interpolate the analyte concentration of each experimental sample using a five-parameter logistic curve-fitting method within the software. Duplicate samples were averaged for final concentration. Analytes that had any sample within the range of the lowest and highest standard were considered in the quantifiable range (31 of 48).

### Single-cell library preparation and sequencing

By modifying a high-throughput single-cell immune profiling assay provided by 10× Genomics (Pleasanton, CA) to incorporate newly designed gene amplification primers, we can now study macaque BCR (22) and TCR sequences. Thus, our data demonstrate the use a key resource in studying immune cell receptors associated with antigen specificity in a macaque pediatric model of HIV-1 infection. Moreover, that we obtained full-length sequences for these receptors provides the opportunity to generate macaque BCR and TCR sequences of interest recombinantly for functional analyses. Libraries were generated from cryopreserved PBMCs using the 10× Genomics Single Cell Immune Profiling 5′ version 1.1 kit. Gene expression (GEX, transcriptome) was made following the manufacturer’s instructions. Single-cell BCR libraries were generated using modifications as previously described (22). TCR libraries were generated using modifications as previously described (https://www.jimmunol.org/content/208/3/762; /https://www.jimmunol.org/content/204/12/3434.abstract).

GEX libraries were sequencing on an Illumina NovaSeq 6000 S4 flowcell using read lengths R1: 26, R2: 91, I1: 8, and I2: 0, and TCR/BCR libraries were sequenced on a NextSeq 500 using read lengths R1: 26, R2: 150, I1: 8, and I2: 0.

### Single-cell transcriptome analysis

Illumina NGS-generated raw data files were processed using the Cell Ranger (version 4.0) single-cell gene expression software provided by 10× Genomics. The Cell Ranger Single Cell Software Suite was used to generate FASTQ files and to perform sample de-multiplexing, barcode processing, reference alignment and single-cell gene counting (78). Reads were aligned to the RM genome (Mmul_10). Matrices of cell barcodes and gene counts generated by Cell Ranger were loaded into Seurat R package (version 4.0) for graph-based cell clustering, dimensionality reduction, and data visualization (79 – 81). SCtransform version 2 was used for normalization and data integration. Automated cell labeling and identification was done using SingleR (82) with the Monaco Immune cell data set (https://pubmed.ncbi.nlm.nih.gov/30726743/) as a reference. SingleR is a program that compares the transcriptional profile of single-cells or clusters in a single-cell experiment to a reference database of transcriptomes generated from primary cell types, allowing automated cell-type identification. The cell types assigned by SingleR can then be confirmed and explored deeper using canonical markers and other multimodal data. Canonical markers used to confirm cell subsets were CD3E, CD4, and CD8 for T cells, CD79B, CD19, MS4A1, CD38, MKI67, FCRL5, IGHM, and CD40 for B cells, and CD14 for monocytes. Canonical markers for other cell subsets were attempted; however, due to the sparse annotation in the macaque reference genome, they were either not found or unannotated, and therefore could not be used. Differentially expressed genes between cell clusters or groups were determined using Seurat by the Wilcoxon rank-sum test. Graphs and plots were generated using the Seurat and ggplot2 R packages.

### Single-cell BCR and TCR sequence analysis

Single-cell rhesus macaque BCR analysis was performed as previously described (22). Single-cell TCR analysis was performed as follows: the inner_enrichment_primers.txt was updated to include the inner enrichment primers used during PCR amplification of the rhesus TCR transcripts (alpha = ATGCACGTCAGAATCCTTGCT, beta = TCTGATGGCTCAAACACAGC, delta = GGGAGAGACGACAATAGCAGGA, and gamma = TGGAGGTTTGTTTCAGCAATGGA). A TCR reference data set for rhesus macaques was imported from imgt using the fetch-imgt command. TCR constant regions for alpha, beta, gamma, and delta TCR chains were taken from Brochu et al. (83). (https://www.ncbi.nlm.nih.gov/pmc/articles/PMC7276939/#DC1) were added to the imgt reference and to facilitate with the reference based assembly done by Cell Ranger. Full-length contigs were annotated with MiXCR (https://www.nature.com/articles/nmeth.3364) using MiXCR’s built-in reference gene libraries of rhesus V, D, J, and C segments, and sequences were assigned with MiXCR into clonotypes.

## Data Availability

The raw sequencing data from our single-cell transcriptome studies were uploaded to the SRA under the archive number PRJNA961687.

## SUPPLEMENTAL MATERIAL

Table S1 jvi.01094-23-s0001.xlsx Viral load measurements.

Table S2 jvi.01094-23-s0002.xlsx List of genes following gene expression analysis of cell populations reported in Figure 4.

Table S3 jvi.01094-23-s0003.xlsx List of genes following gene expression analysis of cell populations reported in Figure 5.

Table S4 jvi.01094-23-s0004.xlsx List of genes following gene expression analysis of cell populations reported in Figure 6.

Table S5 jvi.01094-23-s0005.xlsx Absolute CD4 and CD8 T cell counts.

ASM does not own the copyrights to Supplemental Material that may be linked to, or accessed through, an article. The authors have granted ASM a non-exclusive, world-wide license to publish the Supplemental Material files. Please contact the corresponding author directly for reuse.
